# The neurobiology of misophonia and implications for novel, neuroscience-driven interventions

**DOI:** 10.3389/fnins.2022.893903

**Published:** 2022-07-25

**Authors:** Andrada D. Neacsiu, Victoria Szymkiewicz, Jeffrey T. Galla, Brenden Li, Yashaswini Kulkarni, Cade W. Spector

**Affiliations:** ^1^Duke Center for Misophonia and Emotion Regulation, Duke Brain Stimulation Research Center, Department of Psychiatry and Behavioral Neuroscience, School of Medicine, Duke University, Durham, NC, United States; ^2^Department of Psychology and Neuroscience, Duke University, Durham, NC, United States; ^3^Department of Philosophy, Duke University, Durham, NC, United States

**Keywords:** misophonia, neuroscience, neurostimulation, intervention, review

## Abstract

Decreased tolerance in response to specific every-day sounds (misophonia) is a serious, debilitating disorder that is gaining rapid recognition within the mental health community. Emerging research findings suggest that misophonia may have a unique neural signature. Specifically, when examining responses to misophonic trigger sounds, differences emerge at a physiological and neural level from potentially overlapping psychopathologies. While these findings are preliminary and in need of replication, they support the hypothesis that misophonia is a unique disorder. In this theoretical paper, we begin by reviewing the candidate networks that may be at play in this complex disorder (e.g., regulatory, sensory, and auditory). We then summarize current neuroimaging findings in misophonia and present areas of overlap and divergence from other mental health disorders that are hypothesized to co-occur with misophonia (e.g., obsessive compulsive disorder). Future studies needed to further our understanding of the neuroscience of misophonia will also be discussed. Next, we introduce the potential of neurostimulation as a tool to treat neural dysfunction in misophonia. We describe how neurostimulation research has led to novel interventions in psychiatric disorders, targeting regions that may also be relevant to misophonia. The paper is concluded by presenting several options for how neurostimulation interventions for misophonia could be crafted.

## Introduction to misophonia

Misophonia is a disorder characterized by distress when faced with specific sounds or with the context surrounding such sounds (Brout et al., 2018; Swedo et al., 2021). Sound or visual stimuli, labeled as “triggers,” lead to negative emotional, physiological, and behavioral responses that are more intense than in the general population. Triggers tend to be repetitive and more often than not are generated by another’s human body. Sounds such as chewing, eating, slurping (Vitoratou et al., 2021), throat clearing, and breathing/sniffing are common triggers for misophonia. [See consensus definition for a more comprehensive characterization of trigger sounds (Swedo et al., 2021)]. Once triggered, children and adults with misophonia experience intense distress and have difficulty disengaging from the stimulus (Brout et al., 2018). While triggers may vary from person to person, the typical response involves increased autonomic arousal (muscle tension, increased heart rate, and skin conductance) and self-reported experience of anger, disgust, and anxiety (Siepsiak and Dragan, 2019). This discomfort may translate into behavioral or verbal aggression in the moment, and extreme avoidance behaviors outside of the moment (Swedo et al., 2021).

Misophonia is hypothesized to develop in early adolescence and does not improve with time (Brout et al., 2018; Swedo et al., 2021). More research is needed to better understand the onset of misophonia, with emerging studies suggesting genetics (Sanchez and Silva, 2018; Kılıç et al., 2021), and maladaptive learning (Schröder et al., 2013; Dozier, 2015; Dozier and Morrison, 2017) as possible mechanisms through which this disorder develops. Several studies document that misophonia leads to impairment in functioning and negatively affects interpersonal relationships (Wu et al., 2014; Zhou et al., 2017; Brout et al., 2018; Swedo et al., 2021). The severity of the misophonic reaction depends on the context in which it occurs, the perceived controllability, and the relationship between the individual and the source of the trigger (Swedo et al., 2021). Increased environmental stressors worsen misophonic distress and reduce the ability to downregulate arousal when faced with trigger sounds (Ferrer-Torres and Giménez-Llort, 2021). In addition, misophonic triggers significantly reduce the participant’s cognitive control and ability to engage in goal directed behavior (Daniels et al., 2020).

Misophonia may overlap with other auditory conditions such as tinnitus, hyperacusis (Aazh et al., 2019), autonomous sensory meridian response (ASMR; McErlean and Banissy, 2018; Palumbo et al., 2018; Rouw and Erfanian, 2018), or with psychiatric conditions (Quek et al., 2018; Swedo et al., 2021). One study found a 52.4% overlap with obsessive compulsive personality disorder (OCPD; Cavanna and Seri, 2015) while in other samples this overlap was lower (26%; Jager et al., 2020). Other typical comorbidities are mood disorders (10–48%; Erfanian et al., 2019; Claiborn et al., 2020; Jager et al., 2020), attention deficit and hyperactivity disorder (ADHD; 12%), post-traumatic stress disorder (PTSD; 12–15%; Rouw and Erfanian, 2018; Erfanian et al., 2019; Claiborn et al., 2020), and obsessive compulsive disorder (OCD; 15–21%; Erfanian et al., 2019; Claiborn et al., 2020). Comorbidities with eating disorders have also been reported (10% in one study; Erfanian et al., 2019). One study found that PTSD alone was related to misophonia severity (Rouw and Erfanian, 2018). These studies should be seen as preliminary because they either had small samples (Erfanian et al., 2019), or they asked participants to report what diagnoses they received or thought they had (Claiborn et al., 2020), which may lack accuracy. Nevertheless, research suggests that misophonia is independent from these comorbidities and shouldn’t be diagnosed if the presenting problems are better explained by one of these more established disorders (Swedo et al., 2021). For instance, up to 50% of those who describe misophonic distress do not have any other mental health disorders (Rouw and Erfanian, 2018) indeed, emerging research is painting the picture of a distinct problem that does not fit neatly within a diagnosable disorder (Brout et al., 2018).

## Structure and function of neural regions that may be connected to misophonic distress

One clear way in which misophonia can be distinguished from other disorders is by identifying how exactly this dysfunction translates into aberrant neural function and connectivity. In this section, we introduce regions of interest for misophonia, and describe their broad structure, function, and connectivity patterns. This section is focused on brain regions that have been identified by at least two neuroscientific studies as showing abnormalities in misophonia (see Table 1 and section “The neurobiology of misophonia”). We chose to include review articles coupled with relevant research findings to highlight typical function in these regions in healthy adults. We also highlight, when available, findings of dysfunction in these regions in adults or adolescents who meet criteria for the disorders that most commonly co-occur with misophonia. An exhaustive literature review of all the findings relevant to these regions is beyond the scope of this article. Other regions not detailed below but found in section “The neurobiology of misophonia” may be important areas of intervention and should continue to be investigated.

**TABLE 1:** Summary of brain regions relevant to misophonia, their established function, and the specific alterations in structure and function identified in neuroimaging studies to date.

Brain region	Function	Alterations in misophonia
Insula	Self-awareness, emotional processing, emotional awareness, autonomic homeostasis Anterior insula: autonomic and interoceptive functions, body representation, and emotional experience	Hyper-connectivity to frontal and temporal lobes, V1, V2 at rest; hyperactivation of dorsal anterior insula (bilateral or right) during exposure to trigger sounds; hyper connectivity with DMN, amygdala and hippocampus during misophonic sound exposure
Orbitofrontal/ventromedial prefrontal cortices	vmPFC: Evaluation of risk, downregulation of emotions OFC: decision making, emotional processing, reward valuation, and emotion assignment to sensory input, part of TAO network governing integration of emotional state with cognition and behavior	Hyperconnectivity between lateral OFC and motor cortex (PMv, SMA); increased myelination in vmPFC and OFC-frontal pole and OFC-dlPFC networks
Cingulate cortex	Emotional processing and regulation broadly. ACC: conflict processing, reinforcement learning, motivation, error detection, action selection, management of aggressive behaviors, processing of social pain Rostral ACC: empathy, emotional processing MCC: decision making, executive and motor control, emotion regulation	Right ACC and bilateral MCC hyperactivity during misophonic triggers; hyperconnectivity between MCC and A1 and lateral OFC during trigger sounds; lack of inhibition success-related activity in the PCC
Ventral premotor cortex	Integration of sensory information, calculation of optimal motor response, mirror neurons (e.g., mimicking and predicting intentions of other people)	Hyperconnectivity to A1 and lateral OFC; hyperactivation during misophonic triggers; hyperconnectivity to A2, V2 at rest
Supplementary motor area	PreSMA: Planning complex movements, response selection, conflict resolution, word selection, and decision making SMA: Planning of complex movements, timed deliberate motor execution, emotional empathy	Hyperconnectivity with A1 and lateral OFC during presentation of audio-visual triggers; bilateral hyperactivity during trigger sounds compared to aversive
Superior temporal cortex	Identification and interpretation of sound sources, language and sound processing, auditory attention, interpretation of facial and emotional cues. TPJ: emotional distancing	Hyperactivation during trigger sounds; auditory cortex hyperconnectivity at rest to PMv, SMA, and lateral OFC; hyperconnectivity at rest with the insula; TPJ-right inferior frontal cortex hyperconnectivity at rest.
Amygdala	Emotion processing, decision making in emotional situations, Right amygdala: regulation of negative emotions, detection of dynamic emotional stimuli Left amygdala: processing positive emotions, evaluation of continuous emotional stimuli	Hyperactivity in the left amygdala during trigger sounds compared to aversive stimuli; hypoactivity in left amygdala during aversive stimuli compared to healthy controls; hyperconnectivity with anterior insula during trigger sounds; hyperconnectivity during resting state with the cerebellum; higher myelination on tracts between the amygdala and the occipital cortex; larger gray matter volume in the right amygdala
Dorsolateral prefrontal cortex	Working memory, planning, decision making, feeling of threat-induced anxiety, social perspective taking, theory of mind, deductive reasoning,	Reduced inhibition success-related activation of left dlPFC; increased myelination in tracts connecting OFC to dlPFC;

V1, visual area 1; V2, visual area 2; AIC, anterior insular cortex; A1, primary auditory cortex; A1, secondary auditory cortex; vmPFC, ventromedial prefrontal cortex; OFC, orbitofrontal cortex; DMN, default mode network; TAO, temporo-amygdala-orbitofrontal; SMA, supplemental motor area; PMv, ventral premotor cortex; ACC, anterior cingulate cortex; MCC, midcingulate cortex; TPJ, tempo-parietal junction; and STC, superior temporal cortex.

### Insula

**FIGURE 1:**
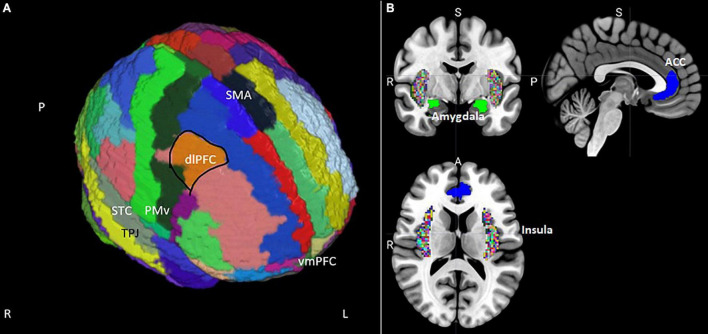
(A) Position of the supplementary motor area (SMA), dorsolateral prefrontal cortex (dlPFC), ventral premotor cortex (PMv), superior temporal cortex (STC), and ventromedial prefrontal cortex (vmPFC) on an MNI template brain segmented using the AAL atlas (Tzourio-Mazoyer et al., 2002). Masks for the dlPFC and vmPFC were extracted from the Mindboggle segmentation (Klein et al., 2005). (B) Coronal, sagittal, and transversal views of subcortical regions relevant to misophonia extracted from Mindboggle and from the FSL Harvard-Oxford sub-cortical structural segmentations. The anterior cingulate cortex (ACC) is blue, the amygdala is green, and the insular cortex is colored rainbow.

The insular cortex (see Figure 1B) can be found in the lateral sulcus, underneath the frontal and temporal lobes (Naidich et al., 2004). It is highly connected with several regions including the frontal, temporal, and parietal lobes, the brainstem, and limbic structures such as the amygdala, thalamus, cingulate gyrus, and basal ganglia (Flynn, 1999). Therefore, the insula is involved in autonomic, self-awareness, and emotional processing functions (Gu et al., 2013). On each side, the insula can be divided into anterior, middle, and posterior sections. Of primary interest to misophonia is the anterior insula, which is a specialized region, involved in autonomic and interoceptive functions (Flynn, 1999). A unique feature of the insula is that it includes a cluster of spindle-shaped von Economo neurons (VENs; Economo, 1926), that are larger than other neurons, and are used for rapid integration of information between the frontal and insular cortices (Gu et al., 2013). The insula is critical for emotional awareness, and is especially involved in the interoceptive “feeling” of the emotion (Gu et al., 2013). The function of the anterior insula has been connected with heart rate and respiration changes, pain, feeling of touch, awareness of temperature, risk, emotional processing, trust, and norm violation (Gu et al., 2013; Droutman et al., 2015). Therefore, this structure is responsible for perceived awareness of one’s physiological state and needs. Insults to the anterior insular cortex (AIC) result in either heightened, or significantly impaired, perceived wellbeing, and emotional awareness. Studies examining the AIC show that it integrates cognitive and motivational information with emotional inputs (Gu et al., 2013). Hyperacussis has also been described in case reports of three individuals with insular damage, denoting that this structure may be critical in the perception and modulation of sound intensity (Boucher et al., 2015).

Degeneration of VENs as well as hypoactivity in the anterior insula have been connected to alexithymia, or deficits in emotional awareness (Bird et al., 2010; Seeley, 2010). Hypoactivity in the bilateral mid-insula has also been connected with depression severity (Avery et al., 2014). Functional hyperactivity of the insula has generally been related to tasks where negative emotional stimuli are elicited in psychiatric disorder (Schienle et al., 2005; Groenewold et al., 2013). One study found hyperactivity in the right AIC when engaging in downregulation of distress in adults who have excessive weight versus matched controls (Steward et al., 2016) as well as in depressed adults (Beauregard et al., 2006). Another study identified hyperactivation in ADHD adolescent boys when compared to typically developing boys during exposure to negative-valenced stimuli (Vetter et al., 2018). In anxiety disorders, it has been shown that anticipating negative stimuli with unpredictable aversiveness is associated with insula hyperactivity (Simmons et al., 2011; Gorka et al., 2014).

Tractography studies observe widespread connections between the insula and the frontal and temporal lobes. Specifically, structural connectivity to superior medial frontal gyri (SMFG), orbitofrontal cortex (OFC), and the auditory cortex (Ghaziri et al., 2017). Furthermore, the anterior insula connects to the anterior cingulate (ACC) and midcingulate cortices (Ghaziri et al., 2017). Studies examining connectivity dysfunction in PTSD patients found increased right AIC-DMN resting state connectivity 2 days after trauma exposure (Wang et al., 2012), and in right AIC-amygdala functional connectivity while listening to trauma reminders (Cisler et al., 2014). A decrease in the functional connectivity between the right AIC and the OFC/ventromedial prefrontal cortex (vmPFC) at rest was found to be uniquely associated with lack of insight in OCD (Fan et al., 2017) and with alexithymia in smoking adults (Sutherland et al., 2013).

### Orbitofrontal/ventromedial prefrontal cortices

The vmPFC (Figure 1A) is located at the bottom of the frontal lobe and is critical for evaluating risk, and regulating emotions, such as fear, by directly influencing the amygdala (Motzkin et al., 2015). The OFC is located just above the orbits, therefore in a similar anatomical location, and encompassing similar Brodmann (BA) areas as the vmPFC (Phillips and Della Sala, 1998). The OFC functions to assist in decision making and emotional processing, and is connected to other prefrontal cortex (PFC) regions associated with decision making (Kahnt et al., 2012). In healthy adults, the OFC has also been associated with assigning a reward value or emotion to primary reinforcers such as taste, texture, or facial expressions (Rolls and Grabenhorst, 2008). In order to make these evaluations, the OFC has connections to the primary taste, inferior temporal, primary olfactory, and somatosensory cortical areas (Rolls, 1996). The OFC also receives input from the amygdala and is responsible for emotional enhancement in memory processing (Rolls, 1996; Kumfor et al., 2013). In one study, atrophy in the OFC was connected in patients with fronto-temporal dementia to then inability to use negative emotions to retrieve memories (Séguin, 2004). Reduced engagement of the OFC has also been connected with difficulty adjusting behaviors after negative feedback in OCD (Cisler et al., 2014) and ADHD patients (Itami and Uno, 2002).

Hyperconnectivity between the OFC and other brain regions can be seen in OCD and obesity (Black et al., 2014; Liu et al., 2022). For example, OFC-ACC/and OFC-caudate hyperconnectivity has been documented in OCD (Liu et al., 2022), and lateral OFC-left dorsolateral prefrontal cortex (dlPFC) hyperconnectivity in obese children when compared to controls (Black et al., 2014). Hyperconnectivity between the vmPFC and striatum, frontal and motor cortices are also connected to OCD, with vmPFC-caudate hyperconnectivity being correlated with OCD severity (Apergis-Schoute et al., 2018).

### Cingulate cortex

The cingulate cortex lies underneath the frontal cortex, in the medial portion of the cerebral hemisphere and is comprised of Brodmann Areas (BA) 23–26 and 29–31 (Stanislav et al., 2013). It mediates the pathway from the thalamus to the hippocampus (Chauhan et al., 2021) and is implicated in emotional processing and regulation, with specific areas (anterior and posterior cingulate cortices) assumed to have differentiated functions. The ACC (Figure 1B) sits at the front of the cingulate cortex, wrapping around the head of the corpus callosum (Monosov et al., 2020). Various examinations implicate the ACC in several aspects of emotion and cognition, including conflict processing, reinforcement learning, motivation, error detection, action selection (Holroyd and Yeung, 2011), management of aggressive behaviors (van Heukelum et al., 2021), and processing of social pain (Eisenberger, 2015). The ACC has also been broken into different subsections, each with differential functions. The rostral ACC (rACC) is closely connected with the amygdala and is thought to play a role in empathy and emotional processing (Singer et al., 2004; De Brito et al., 2009). For example, loss of white matter tracts connecting the rACC with the amygdala diminishes the ACC’s ability to inhibit amygdala activation, leading to increased fear responses seen in PTSD (O’Doherty et al., 2018). The dorsal ACC (dACC), also labeled the midcingulate cortex (MCC; Stevens et al., 2011), is connected to the dlPFC, supplementary motor area (SMA), supramarginal gyrus, and insula revealing its involvement in social cognitive and motor processes (Overbeek et al., 2021; Tuovinen et al., 2022). The MCC was found to mediate decision making, executive control (Mattavelli et al., 2022), and emotion regulation (Li et al., 2014) with increased activation in the anterior MCC being associated with decreased negative emotion (Stevens et al., 2011). Increased ACC activity during a task has been associated with improved emotion regulation (Tang et al., 2016).

### Ventral premotor cortex

The ventral premotor cortex (PMv; Figure 1A) is located within the frontal cortex overlapping with the Brocca region and including mirror neurons involved in mimicking and predicting the intentions of others (Binkofski and Buccino, 2006). The PMv is a receptacle for multi-sensory inputs including tactile, auditory, and visual, and is highly connected with adjacent premotor cortical regions (Boussaoud, 2001; Pardo-Vazquez et al., 2009). Time and sensorimotor integration of inputs have been causally connected to the PMv using an inhibitory neurostimulation paradigm (Ruspantini et al., 2011). In synchrony with adjacent areas, the PMv calculates the optimal motor response given learned consequences of several possible options (Pardo-Vazquez et al., 2008; Lemus et al., 2009). Individuals with PMv impairment have difficulty adjusting motor responses based on feedback (Berthier et al., 2017).

### Supplementary motor area

The SMA (Figure 1A) of the brain is located on the medial surface of the cortex anterior to the pre central sulcus and is divided into two sub-regions (Kaas and Stepniewska, 2002). The preSMA connects predominantly with areas that are fundamental to a wide spectrum of functions such as planning complex movements, response selection, conflict resolution, word selection, and decision making (Tremblay and Gracco, 2009). The SMA proper occupies a significant portion of the superior frontal gyrus, taking up roughly one third of its territory, with a primary function of planning complex movements and deliberate motor execution (Tremblay and Gracco, 2009). Lesions within the SMA have resulted in an impaired ability to produce self-initiated or unique motion; however, motions that have a strong conditional response based upon memory are maintained as they may be generated largely by using previously associated sensory cues (Kaas and Stepniewska, 2002).

The rich connections of the SMA to the somatosensory regions allow this region to function as more than simply a point of translation of sensory stimuli into motor activity (Kaas and Stepniewska, 2002). SMA neurons receive sensory inputs and interpret them for the purpose of planning and controlling the timing of a motor response so that movement is deliberate and planned and not simply reflexive in nature. In connection with the aMCC and the left anterior insula, SMA has also been hypothesized to underline the experience of empathy for other people’s fear or disgust emotions (Fan et al., 2011). Examinations of the SMA in psychopathology show deficits in activation and in integration of sensory input in ADHD and other developmental disorders (Piek and Dyck, 2004; Mostofsky et al., 2006). Therefore, unlike other brain regions, hyperactivity and hyperconnectivity in this region is uncommon in psychopathology.

### The superior temporal cortex

The superior temporal cortex (STC; Figure 1A) comprises roughly 17% of the total cerebral cortex in humans and includes areas with auditory, olfactory, vestibular, visual, and linguistic functions (Kiernan, 2012). The predominant role of the STC is to identify the source of a sound and interpret it as human, dog, car, etc. (Kiernan, 2012). The STC can be divided into two regions with some functional overlap: the superior temporal gyrus (STG) and the superior temporal sulcus (STS). Likely most relevant to misophonia, the STG is fundamental to human understanding of language and sound. It provides the ability to analyze facial expressions and changes in terms of the inherent emotional messaging (Bigler et al., 2007). Additionally, the STG has a functional role in auditory processing within the context of ambient noise. Specifically, this region becomes more active in social situations when a person strains to hold conversation amid a background information (Vander Ghinst et al., 2016). The tempo-parietal junction (TPJ), which captures part of the STG, has also been connected with the ability to engage successfully in emotional distancing if the context requires it (Powers et al., 2020a,b; Powers and LaBar, 2019). Hyperactivity of the STG during exposure to trauma has been one of the brain abnormalities distinguishing adults diagnosed with PTSD from controls (Lanius et al., 2002).

### Amygdala

The amygdala (Figure 1B) is a key structure in the brain for processing emotions. It lies in the medial temporal lobe in front of the hippocampus and is a part of the limbic system. Extended research has found strong connectivity between the amygdala and the cingulate cortex, OFC, insular cortex, dlPFC, and parahippocampal gyrus (Stein et al., 2007). The amygdala also interacts with the vmPFC when we engage in affective decision-making. The hypothesized role of the amygdala is to assess the affective components of the situation and the of the vmPFC to lead to a judgment based on amygdala inputs (Shenhav and Greene, 2014). Differences between the roles of the right and left amygdala have been identified and extensively researched. In brief, the right amygdala is naturally larger than its left counterpart in healthy adults, resulting in asymmetrical lateralization (Murphy et al., 1987). It uniquely connects to the dorsomedial (dm)PFC, a neural pathway that has been connected to the regulation of negative emotions (Baeken et al., 2014). On a behavioral level, the right amygdala is more engaged in processing negative emotional stimuli as opposed to the left amygdala which is involved primarily in processing positive emotional stimuli (Yoshimura et al., 2009). More prominent involvement in habituation (Wright et al., 2001) and fear conditioning (Baker and Kim, 2004) led to the hypothesis that the right amygdala is part of a system that detects dynamic emotional stimuli, while the left amygdala may be more involved in evaluating a continuous stimulus. Higher volume in the left amygdala has also been connected with enhanced empathy (Goerlich-Dobre et al., 2015). The pervasive amygdala dysfunction found in psychopathology tends to be associated with hyperactivation or atypical connectivity of the right amygdala (Abercrombie et al., 1998; Chen et al., 2004; Gilboa et al., 2004; Kleinhans et al., 2010; Spoletini et al., 2011; Pico-Perez et al., 2017; Thorsen et al., 2018; Picó-Pérez et al., 2019) or insufficient activation in the left amygdala (Thorsen et al., 2018).

## The neurobiology of misophonia

Initial conceptualizations of misophonia hypothesized neural hyper-connectivity between auditory and limbic systems (Jastreboff and Hazell, 2008) based on prior examinations of heightened noise sensitivity which found volumetric enhancements in left auditory areas, bilateral hippocampus, and right anterior insula (Kliuchko et al., 2018)^1^. Therefore, initial examinations of the neurobiology of misophonia have focused on the auditory and emotional neural networks. In a preliminary investigation, 10 misophonic adults, and 7 matched healthy controls were exposed to 25 s audio-video clips of neutral, aversive, or misophonic trigger sounds (San Giorgi, 2015). Participants with other mental health disorders were excluded. Increased activity in the left amygdala was found for those diagnosed with misophonia versus controls when being exposed to trigger versus aversive videos (San Giorgi, 2015). This preliminary finding suggested that emotional neural networks, rather than auditory over-responsivity, might be key in understanding misophonia. Specifically, given the hypothesis that the left amygdala may be more involved in evaluating a continuous stimulus as negative, trigger sounds may be overly identified as negative experiences in misophonic contexts. Furthermore, given that the left amygdala tends to under perform in the majority of psychiatric disorders, hyperactivation in misophonia points to a unique neural signature for this disorder.

Kumar et al. (2017) examined 20 adults diagnosed with misophonia and 22 controls (non-misophonic) using a functional neuroimaging paradigm. Participants had to report being bothered by sounds of eating, breathing and chewing regardless of whom the person engaging in these behaviors was. Control participants were briefly exposed to misophonic triggers and only enrolled if they did not describe a misophonic response to these sounds. No additional clinical characterization of the two groups was performed. Exposing participants to 15 s audio clips of misophonic sounds led to significantly higher activation in the bilateral AIC in adults diagnosed with misophonia versus controls. The researchers concluded that trigger sounds may have been perceived with heightened salience in those who experience misophonia. This enhanced activation mediated increases in heart rate (HR) and galvanic skin response in adults with misophonia. Furthermore, during trigger sounds alone, the AIC (especially in the left hemisphere) had enhanced functional connectivity with core parts of the default mode network (DMN; the vmPFC, posteromedial cortex), with the hippocampus, and with the amygdala. This hyperconnectivity suggests that individuals with misophonia have difficulty disconnecting default mode memories of similar contexts and thoughts from the active situation, increasing the salience of the experience (Kumar et al., 2017). The enhanced connectivity to the amygdala is also seen in PTSD during trauma exposure (Cisler et al., 2014), suggesting that perception of trigger sounds may be akin to trauma exposure.

Using a similar paradigm but changing the stimuli to include both video and audio, Schröder et al. (2019) compared 21 adults with misophonia with 23 controls. Unlike Kumar et al. (2017), this study excluded participants with psychiatric comorbidities (e.g., anxiety, mood, or substance use disorders) and did not find differences between groups when comparing misophonic (lip smacking, loud breathing) with aversive triggers. Nevertheless, when comparing misophonic with neutral sounds, the study found significant higher activation of the right insula, ACC, and STC in adults with misophonia versus controls. Increased heart rate and self-reported anger, disgust and sadness in response to misophonic triggers also differentiated adults diagnosed with misophonia from controls. Furthermore, repeated exposure to the same triggers amplified the salience network activity which the authors hypothesized indicated a conditioned response, augmented by enhanced vigilance (Schröder et al., 2019).

A novel finding in this study is the increased ACC function during symptom provocation which may suggest that adults diagnosed with misophonia may engage in enhanced emotion regulation during perception of misophonic trigger sounds (Schröder et al., 2019; Cerliani and Rouw, 2020). Based on the function of the ACC presented in the last section, is also possible that increased ACC activation during misophonic triggers can be a marker of higher perceived social pain (or rejection) when sounds produced by another person are experienced. In addition, hyperactivation of the STC during trigger sounds in misophonia may suggest that misophonic sounds are involuntarily brought into the forefront of the context, enhancing their salience.

Cerliani and Rouw (2020) compared 19 participants with misophonia and 20 controls. Twelve-second audio-visual stimuli that were either neutral, aversive, or misophonic triggers were presented during an fMRI paradigm. Each stimulus was preceded by a 2 s text description. Significantly higher brain activity was found during perception of triggers versus aversive sounds in misophonic adults versus controls in the SMA, MCC, visual cortex (V1/V2), and right ventrolateral premotor cortex (PMvl), including the right anterior insula. In addition to enhanced activation, researchers also found hyperconnectivity between MCC/SMA/PMvl with the primary auditory cortex (A1), and the lateral OFC, a signature that appears to be unique to misophonia. Interestingly, these differences were not found when comparing trigger sounds with neutral sounds.

The areas of enhanced activation are responsible for planning motor behavior and may represent the misophonic adult’s preparation to avoid or physically react to the sound. The authors hypothesize that the misophonic reaction is not a direct auditory-limbic response, rather a more complex process mediated by higher order processes. The important role of the lateral OFC connects misophonia to difficulties with behavioral inhibition and with learning how to adjust responses to misophonic triggers based on context. Specifically, adults with misophonia may have difficulty reassessing the unnecessary negative response associated with this particular type of innocuous auditory stimuli (Cerliani and Rouw, 2020). This finding supports the high co-occurrence between misophonia and compulsive disorders. Concerning the SMA, the heightened connectivity with the auditory cortex can suggest higher salience of the auditory input when determining upcoming motor action. The higher SMA activation during trigger versus aversive sounds may suggest a stronger impulse to escape, or to overly identify with the person engaged in the actions that yield misophonic sounds.

Concerning the AIC, two studies showed hyperactivity during exposure to trigger sounds in the absence of regulation instructions (Kumar et al., 2017; Schröder et al., 2019). However, in one study when the unpredictability of the trigger may have been reduced by announcing the type of sound with a written description, the hyperactivity seen in the insula was reduced (Cerliani and Rouw, 2020). This may suggest that reducing ambiguity may lessen the insula reactivity, although a direct comparison is needed to support such a conclusion. Therefore, it is possible that the unpredictability of how a misophonic situation might unfold (i.e., how much worse the trigger sound will get), negative emotions associated with the sounds, and automatic engagement in maladaptive emotion regulation may be responsible for the insula hyperactivity seen in misophonia. It is also possible that hyperactivity in the insula in misophonia may be connected to deficits in attention (Eijsker et al., 2019).

Eijsker et al. (2019) administered a stop signal task to 25 adults with misophonia and 25 matched controls. Participants were excluded if they met criteria for bipolar I, psychosis, substance use disorders, autism, as well as other neurological conditions. The majority of the sample had no comorbidities except one case with ADHD, and one case with borderline personality disorder. There were no differences in successful engagement in the task between the two groups. Nevertheless, misophonic adults evidenced reduced inhibition success-related activation of left dlPFC and heightened activation in this region during correct going trials when compared to controls. Furthermore, misophonic adults only activated the SMFG less during inhibition success compared to failure, suggesting that feedback that may normally be passed to the insula adjusts its activity may be missing or insufficient. Controls only showed inhibition success-related activity in the posterior cingulate cortex (PCC).

The authors interpret these findings as suggesting strategic delay of responses in misophonic adults who may prefer accuracy over speed (e.g., perfectionism) during this task coupled with overly negative self-reflection in response to failure and an inability to adjust strategy employed in response to success and failure information. Others have also suggested that perfectionism may be at play within misophonic patients (Jager et al., 2020; Natalini et al., 2020), using self-reports, personality assessments, and clinical examples, although the research data is mixed (Szykowny, 2020). One recent experimental study highlights that disproportionate attention to detail coupled with cognitive inflexibility (which can also be construed as perfectionism) is more prevalent in misophonic versus non-misophonic adults (Simner et al., 2022).

Resting state connectivity studies also offer insight into brain differences that are related to misophonia. In a separate assessment of the sample presented above, Eijsker et al. (2021b) examined differences at rest between misophonic adults and matched controls. A multivariate connectivity analysis with the bilateral amygdalae as seed regions showed hyperconnectivity during resting state with the cerebellum for patients when compared to controls. Patients also showed stronger connectivity within the right frontal cortex (IFC) and TPJ at rest. The authors hypothesize that these abnormalities reflect a tendency to enhance sensory processing of emotional information and may lead to reflex-like behaviors. Enhanced connectivity between the TPJ and the frontal cortex at rest could also be connected with difficulty recruiting this area for successful distancing from distressing noise in misophonic contexts (Powers and LaBar, 2019).

Taken together, these studies highlight the neural networks involved in misophonia and *do not* support the hypothesis that misophonia is simply a noise sensitivity problem. Rather, these studies suggest that brain networks involved in emotion, salience, attention to detail, and cognitive flexibility may display aberrant function and connectivity in adults with misophonia (See Supplementary Table 1 for specific locations for neural differences found in the reviewed studies). Evidence supports amplified physiological reactivity to misophonic cues that seems to be connected to emotion much more than to heightened responsivity of the auditory cortex. Similar to other clinical disorders, prefrontal areas may be hypoactive, or connected to these limbic structures in a dysfunctional way (Kumar et al., 2017) leading to difficulties downregulating this exaggerated arousal response. The specific ways in which the insula connectivity is altered and the presence of hyperactivity in motor areas during symptom provocation highlight that *a unique neural signature of misophonia exists.* Small sample sizes and differences in inclusion/exclusion criteria suggest the need for replication for these findings in more tightly controlled samples, including comparisons with clinical not just with healthy controls. The wide range of functional differences found from controls in different studies also points to the necessity of a large trial that can clarify with more accuracy which of these candidate regions shows the most robust evidence for dysfunction in misophonia. Interestingly, all studies point toward hyperfunction in motor areas as being connected to misophonic responses.

Along these lines, Kumar et al. (2021) proposed a new mechanism for misophonia that involves the activity of mirror neurons found in premotor areas. Using a resting state fMRI (rs-fMRI) paradigm, the team showed that function in the right secondary auditory and the PMv cortices are significantly more correlated at rest in 17 adults with misophonia than in 20 controls. A similarly increased functional connectivity at rest was found between the right secondary visual cortex (V2) and the PMv. The right anterior insula also demonstrated heightened functional connectivity at rest with the right V2 and the left primary visual cortex (V1) in adults diagnosed with misophonia versus controls. When exposed to 15 s audio clips, 19 participants with misophonia showed again a stronger connectivity between the auditory cortex and the PMv regardless of the sounds being played. The PMv demonstrated higher activity in misophonic adults when compared to controls for trigger sounds only, and the magnitude of activation in this area correlated with the self-reported distress induced by the sound. The authors identify that the area that demonstrates hyperactivity within the PMv is responsible for engaging in or observing mouth and lip movements. They conclude that this pattern indicates hyperactivity of mirror neurons in areas responsible for orofacial actions.

This neural mirroring may involve behavioral mirroring, likely done with enhanced awareness, and inability to disconnect or distract from this activity (Kumar et al., 2021). Given that the PMv is also activated when observing lip movements of others (Buccino et al., 2001), it is possible that misophonia includes an inability to disengage from sensory cues related to others orofacial movements. Given the function of the PMv, this pattern of results may also suggest over-preparedness for the reactions of others and higher importance given to the motor movements involved in trigger sounds versus other sensory inputs. This hypothesized mechanism doesn’t explain why some trigger sounds, like clicking, elicit misophonic reactions (Hansen et al., 2021), but it offers a comprehensive explanation for neuroimaging findings presented in the literature thus far. A possibility exists that different subtypes of misophonia may exist, or that different mechanisms through which the misophonic reaction is triggered may be at play.

### Structural abnormalities

The first structural abnormality related to misophonia was reported by Kumar et al. (2017) who found higher myelination in the vmPFC. Increased myelination of the vmPFC can also be seen in monkeys who are exposed to early mild life stressors and learn to cope with such stressors (Katz et al., 2009), suggesting that this brain difference may be a marker of resilience. More recent studies failed to replicate this finding when using voxel-based morphometry along with structural MRI; nevertheless, new findings emerged (Eijsker et al., 2021a,b). When compared to controls (*n* = 25) patients with misophonia (*n* = 24) may have larger gray matter volume in the right amygdala (Eijsker et al., 2021b), a finding believed to be connected to the increased emotional reactivity when being exposed to trigger sounds. The same team also showed that adults with misophonia may have greater white matter volumes in the left frontal cortex (the left inferior fronto-occipital fasciculus, and the left body of the corpus callosum; Eijsker et al., 2021a). This finding again points toward a unique neural characteristic in misophonia, given that the majority of mental health dysfunctions have been connected to *lower*, not higher, white matter volumes (Thomason and Thompson, 2011). Patients may also have lower diffusivities, which reflects higher myelination. The affected regions involve tracts connecting the amygdala with the occipital cortex, and the OFC with the frontal pole and the dlPFC (Eijsker et al., 2021a). The authors interpret these findings as possibly underlying processes responsible for disengaging attention away from the aversive stimulus (Eijsker et al., 2021a). The regions affected are also involved in emotional empathy and recognizing facial emotions (Philippi et al., 2009; Oishi et al., 2015), processes that have not been studied in misophonia but which may play a role.

### Future directions for the neuroscience of misophonia

The rapid advancements in understanding the neuroscience behind misophonia are promising. One drawback of the current literature is the little overlap in findings, despite similar paradigms. Therefore, one important future direction may be to examine differences in much larger samples, using more stringent controls for comorbidities. In addition, employing tasks that elicit activation in the insula or the PMv in misophonic patients, in the absence of misophonic triggers, may shed light onto the specificity or generalizability of the observed dysfunction. For example, examining differences in empathy or emotional awareness in adults with and without misophonia (Philippi et al., 2009; Oishi et al., 2015), may provide additional insight into insula hyperactivation.

Future research should also examine neural differences between misophonia and clinical controls during emotion regulation. The rich body of literature of neural underpinnings and plasticity of emotion regulation (Gross, 2013; Powers and LaBar, 2019) may provide important additional avenues for misophonia interventions. An additional important future direction is to examine the developmental trajectory of neural changes in misophonia. Imaging studies in misophonic children do not yet exist, and longitudinal examinations that show the trajectory of dysfunction over time are also lacking.

Neurostimulation can be a helpful tool in understanding causality and development. Therefore, paradigms that attempt to temporarily enhance insula activity and measure sensitivity to triggers in non-misophonic adults may answer questions about misophonic mechanisms. Temporarily altering the function of other brain regions related to misophonia could also provide future insight into the causality of these dysfunctions in relation to misophonia distress.

Last, but not least, misophonic triggers are context specific. In other words, the sound must elicit specific visual imagery, and must come from a specific set of people in order to trigger a misophonic reaction. Neuroimaging examinations that separate these different components of the trigger experience are also needed.

## The neuroscience of overlapping disorders

Knowledge of neurological dysfunction that can be seen in comorbid conditions may broaden our understanding of the neurobiology of misophonia (see Table 2). We chose to focus this section of the review on psychiatric comorbidities, while acknowledging that there is much to learn from misophonia’s overlap with tinnitus, ASMR, or hyperacusis [see McGeoch and Rouw (2020) for an example]. Our review is restricted to psychiatric comorbidities because the solutions proposed for neuroscience-based interventions were primarily developed for psychiatric conditions. We focus on OCPD/OCD, mood disorders, ADHD, and PTSD because several papers have supported their co-occurrence with misophonia, and the rates of overlap appear to be over 10% (Cavanna and Seri, 2015; Quek et al., 2018; Rouw and Erfanian, 2018; Erfanian et al., 2019; Claiborn et al., 2020; Jager et al., 2020; Swedo et al., 2021). Other comorbidities may also occur and have relevance to the neurobiology of misophonia. Nevertheless, existing data either point to low co-occurrence [e.g., autism was reported by 3% of participants with misophonia in a large study (Claiborn et al., 2020)], or there needs to be replication to ascertain the relevance of a comorbidity to misophonia [e.g., for eating disorders (Erfanian et al., 2019)].

**TABLE 2:** Summary of alterations in brain regions that are relevant to misophonia in disorders who have been shown to have comorbidity with misophonia.

	*OCPD*	*OCD*	*MDD*	*Bipolar disorder*	*ADHD*	*PTSD*
*Insula*	Increased higher amplitudes of low-frequency fluctuation in left insula at rest; smaller gray matter volume	Hyperactivity during symptom provocation; during exposure to pictures eliciting disgust and fear; increased connectivity with the dmPFC; hyperactivity in the right anterior insula during error processing and hypoactivity during inhibitory control	Hyperactivity during exposure to negative stimuli and during emotion regulation, and hypoactivity during exposure to positive stimuli	Reduced volume as a precursor of development of this disorder; deactivation during cognitive interference trials and altered connectivity with the DMN	Hyperactivity when presented with negative stimuli distractors; right anterior insula function connected to emotion dysregulation in ADHD	Hypoconnectivity with frontal regions, hyper-connectivity with DMN and periaqueductal gray at rest; reduced volume; functional hyper-connectivity with amygdala during trauma cues
*OFC/* *vmPFC*	Smaller OFC volume	Decreased activation of the left OFC during symptom provocation; hypoactivity during inhibitory control	Hyperactivity in the OFC when presented with positive stimuli; disrupted functional connectivity between OFC and nucleus accumbens	Hypoactivity in the OFC during emotion regulation	Reduced OFC activity when processing reward	Decreased mPFC volume and inverse correlation between responsiveness of the mPFC and symptom severity; decreased gray matter volume in right PFC
*Cingulate cortex*	Smaller gray matter in the cingulate cortex	Decreased activation in the MCC during symptom provocation; decreased gray matter volume in the ACC; hyperactivity in the dorsal ACC during error processing, and ventral ACC hypoactivity during inhibitory control	Hyperactivity in the ACC when presented with negative stimuli or with facial expressions/hypoactivity when exposed to positive or non-facial stimuli	Deactivation during cognitive interference trials in the MCC	Hypoactivity in the dACC when learning verbal fear cues	
*PMv*			Decreased cortical thickness in the left premotor cortex predicts treatment response		Reduced premotor cortex surface area in ADHD boys; hypoactivity when ignoring distractors	Decreased premotor cortex volume
*SMA*		Increased connectivity between the caudate the SMA at rest; hyperactivity during error processing	Functional connectivity with nucleus accumbens is positively correlated with cognitive impairment		Underperformance during neurocognitive tasks	Reduced gray matter volume
*STC*		Increased rest connectivity between caudate and superior and middle temporal gyrus			Compensatory recruitment during response inhibition.	
*Amygdala*	Smaller volume	Hyperactivity during symptom provocation	Hyperactivity when presented with negative stimuli/hypoactivity to positive stimuli	Hyperactivity when presented with emotional stimuli and during regulation; reduced volume	Hyperactivity when learning to discriminate aversive stimuli *via* verbal instruction	Hyperactivity connected to symptom severity
*dlPFC*	Smaller gray mater volume in the prefrontal cortex	Hypoactivity during a planning task	Less activation when exposed to negative stimuli	Hypoactivity during regulation	Hypoactivity in the left dlPFC during working memory and selective motor response inhibition tasks; hypoactivity in the right dlPFC during response inhibition tasks	Decreased gray matter volume in right dlPFC

OCPD, obsessive compulsive personality disorder; OCD, obsessive compulsive disorder; MDD, major depressive disorder; ADHD, attention deficit and hyperactivity disorder; PTSD, post traumatic stress disorder; PFC, prefrontal cortex; dACC, dorsal anterior cingulate cortex; MCC, midcingulate cortex; OFC, orbitofrontal cortex; vmPFC, ventromedial prefrontal cortex; PMv, ventral premotor cortex; SMA, supplementary motor area; STC, superior temporal cortex; dlPFC, dorsolateral prefrontal cortex; and DMN, default mode network.

### Obsessive compulsive personality disorders/obsessive compulsive disorder

Much of the neural mechanisms and dysfunctions in OCPD are difficult to distinguish from OCD. OCD is characterized as a chronic compulsive disorder, while OCPD is a behavioral disorder defined by immoderate perfectionism (Gordon et al., 2013). Emerging examinations of OCPD neural dysfunction highlight altered activity during resting state in the bilateral caudate, left insula, and left medial SFG areas when compared to healthy controls (Lei et al., 2020). These findings highlight higher engagement in self-perception and future planning at rest, which may play into the perfectionistic tendencies seen in this disorder. OCPD patients also have decreased gray matter volume in the prefrontal, cingulate, and insular cortices (Reetz et al., 2008). The decreased gray matter volume of these areas most obviously affects decision-making and correlates with anxious tendencies such as avoidance behavior (Reetz et al., 2008). Furthermore, OCPD patients have decreased amygdala, hippocampus (Gurok et al., 2019), and OFC volumes, and higher volume in the thalamus (Atmaca et al., 2019), suggesting difficulties with memory, emotional processing, and conscious attention. An fMRI study showed greater functional connectivity within the precuneus, a region that controls memory retrieval and manipulation, in OCPD adults when compared to healthy controls (Coutinho et al., 2016). Thus, the neural dysfunction seen in OCPD does not overlap with any of the current findings of neural dysfunction in misophonia, although direct comparisons are warranted. Interestingly, OCPD is characterized by reduced amygdala volumes, with in misophonia amygdala volumes may be increased.

Similar to OCPD, meta-analytic findings in OCD highlight increased activation of the right caudate, putamen, and insula as well as decreased activation of the left OFC, caudate, and MCC when compared to controls (Yu et al., 2022). During resting state, it has also been found in patients with OCD that connectivity within the dmPFC-thalamus-caudate loop is decreased, while connectivity between the caudate and the superior and middle temporal gyrus, middle and inferior occipital gyrus, and SMA is increased (Chen et al., 2016). These neural findings have been associated with disruptions in processing during distress, dysfunctional memory formation, and impairments in cognitive and behavioral regulation. Increased activity in the insula (Schienle et al., 2005) and connectivity with the dmPFC was found as well (Beucke et al., 2014), highlighting the insula’s critical role in emotional processing and feelings of disgust often seen in OCD. Symptom provocation in OCD also shows hyperactivity in the left amygdala (Simon et al., 2014). Studies examining how patients with an OCD diagnosis learn from errors and inhibit behaviors show a hyperactive error processing mechanism and an impaired ability to engage in inhibitory control (Norman et al., 2019). These alterations have been connected with hyperactivity in the dACC, SMA, pre-SMA, right anterior insula, and anterior lateral PFC during error processing, and hypoactivity in the rACC, OFC, and right anterior insula during inhibitory control (Norman et al., 2019). Structural examinations find that patients with OCD have smaller gray matter volume in the ACC compared to controls, suggesting deficits with motor control and visuospatial function (Peng et al., 2012).

Unlike with OCPD, there is some overlap in neural dysfunction between OCD and misophonia. Specifically, hyperactivity in the insula and amygdala is seen during symptom provocation in both disorders as well as hyperconnectivity between the insula and frontal regions, although the specific aberrant connectivity is different between OCD (dmPFC-insula) and misophonia (vmPFC-insula). Hyperactivity in the ACC is found in both disorders, during trigger sound exposure in misophonia and while engaging in an error processing task in OCD. It would be interesting to examine whether exposure to disgust and fear, as well as error processing ’lead to hyperactivity in the insula in misophonia, like they do in OCD. In other networks, OCD and misophonia neuroimaging results diverge. For example, the function of the OFC shows impairment in OCD, while in misophonia the connectivity of the OFC seems to be primarily affected. During symptom provocation, the MCC is hyperactive in misophonia and hypoactive in OCD. Inhibitory control shows dysfunction in the ACC in OCD and the PCC in misophonia. The function of SMA is differentially affected in both disorders: in misophonia trigger sounds lead to SMA hyperactivity, while in OCD error processing tasks lead to SMA hypoactivity. The STC shows altered connectivity at rest in both disorders, but with very different brain regions. Therefore, misophonia is unlikely to be a variant of OCD given that the neuroscientific results point primarily to differences and not to overlapping patterns.

### Mood disorders

There has been considerable effort to characterize neural dysfunction in mood disorders in the recent years. Summaries of this literature point to decreased activation in the dlPFC during exposure to negative stimuli and sustained activation in the amygdala in adults diagnosed with major depressive disorder (MDD) when compared to controls (Groenewold et al., 2013). Hyperactivation in the OFC when presented with positive stimuli and hyperactivity in the ACC (Groenewold et al., 2013) [particularly the subgenual ACC (sgACC)] (Gray et al., 2020) and insula when being exposed to negative stimuli (Groenewold et al., 2013) or when engaging in emotion regulation (Beauregard et al., 2006), are also markers of depression. Severity of depression and cognitive dysfunction has been associated with alterations in functional connectivity between the nucleus accumbens, the OFC, ACC, SMA, and caudate (Gong et al., 2017). Decreased thickness in the left premotor cortex is characteristic of depression, and indicative of a positive response to antidepressants 8 weeks later (Liu et al., 2021).

The markers of psychopathology in bipolar disorder are somewhat different. During emotional processing and regulation, those who meet criteria for bipolar disorder show hyperactivity in the amygdala, hypoactivity in the ventrolateral prefrontal cortex and OFC, and decreased connectivity between these regions when compared to healthy controls (Phillips and Swartz, 2014). Reduced insula and amygdala volumes may be a precursor for bipolar disorder (Bechdolf et al., 2012). Furthermore, during cognitive interference trials when compared to controls, bipolar adults evidence deactivation in the anterior insula and the ACC with altered connectivity with the DMN (Ellard et al., 2019). The hypoactivity in the dlPFC remains a consistent marker of both unipolar and bipolar mood disorders (Townsend and Altshuler, 2012). Structural studies find reduced gray matter volume in the right SMA in both MDD and bipolar adults when compared to clinical and non-clinical controls (Chang M. et al., 2018).

Just like with OCD, there is overlap between misophonia and mood disorders in the neural dysfunction seen during symptom provocation, which in mood disorders takes the form of exposure to negative emotional stimuli. In both groups, symptom provocation leads to hyperactivity in the amygdala and insula, as well as in the ACC. Interestingly, exposure to positive stimuli leads to hypoactivity in these regions, a phenomenon that would be interesting to test in misophonia also. The OFC and SMA function and connectivity are altered in both mood disorders and misophonia, but in very different ways, pointing toward divergence between these disorders. Emotion regulation tasks within mood disorders also lead to a pattern of aberrant function and connectivity, but have not yet been studied in misophonia. Thus, examinations of emotion regulation and positive emotional processing are warranted, although they are unlikely to alter the current conclusion that misophonia has a different neural signature than mood disorders.

### Attention deficit and hyperactivity disorder

Meta-analyses of neuroimaging findings aimed to capture dysfunction in ADHD found decreased activity in the bilateral SFG and left dlPFC during working memory tasks as well as in the bilateral inferior frontal gyri, right SFG, and right dlPFC during response inhibition tasks (McCarthy et al., 2014). In addition, in tasks testing selective motor response inhibition, less activation in the left dlPFC and right caudate was found (McCarthy et al., 2014). The decreased activity in frontal regions correlate to behavioral symptoms of decreased working memory capacity, inhibitory control, self-regulation, and impulsivity control (McCarthy et al., 2014). Dysfunction in reward processing has also been connected to ADHD, specifically to hypoactivity in the OFC when compared to controls (Van Den Heuvel et al., 2005). Underperformance in the SMA and the basal ganglia during neurocognitive tasks, and poor deactivation in the DMN when switching to response inhibition have also been found to differentiate ADHD patients from controls (Albajara Sáenz et al., 2019).

Similar to other misophonia comorbidities, in ADHD when compared to controls, exposure to negative stimuli leads to higher activation in the insula (Vetter et al., 2018). In a rigorous analysis of over 140 participants, investigators concluded that the right anterior insula is likely the hub for altered emotion regulation function in ADHD young adults (Viering et al., 2021). Another study examined learning of fear cues in ADHD and found diminished activation in the dACC when unlearning an instructed fear cue as well as increased activation in the amygdala when being exposed to a neutral cue in the absence of fear instructions (Maier et al., 2014). The authors interpreted these findings as impairments in processing of verbally aversive information in ADHD. Unlike other comorbid disorders, dysfunction in motor areas has been associated with ADHD impairments. In one study, boys diagnosed with ADHD had reduced premotor cortex areas when compared to typically developing children (Dirlikov et al., 2015). Dysfunction in the premotor cortex was also evidenced by an fMRI study showing insufficient recruitment of this area when trying to suppress distractions (Vaidya et al., 2005). When attempting response inhibition children with an ADHD diagnosis recruited the right STC unlike comparison children, who recruited a front-striatal network for this task (Vaidya et al., 2005).

Alterations in the dlPFC function during response inhibition tasks as well as hyperactivity in the insula when presented with upsetting stimuli appear to be commonalities between misophonia and ADHD. The STC is hyperactive in misophonia during trigger sounds, and appears to be recruited as a compensatory mechanism to handle response inhibition in ADHD. These findings may point to overlapping mechanisms of these disorders, although a direct comparison is needed to examine this hypothesis. On the other hand, alterations in other brain regions appear to be very different between misophonia and ADHD. For example, difficulties ignoring distractors are connected to PMv hypoactivity in ADHD, while trigger sounds lead to PMv hyperactivity in misophonia. OFC function appears to be altered in ADHD at least in one domain, while in misophonia evidence primarily points toward dysfunctional connectivity. Thus, misophonia and ADHD are likely to be very distinct disorders.

### Post-traumatic stress disorder

Post-traumatic stress disorder is a disorder that develops in a subset of children and adults who experience a traumatic event (Kessler et al., 2005). A review study conducted in 2016 summarized a decade’s worth of literature regarding the neurobiological basis for PTSD and concluded that the amygdala, the mPFC, and the hippocampus played important roles in the development and maintenance of this disorder (Shin et al., 2006). Specifically, this review concludes that patients with PTSD evidence a heightened amygdala responsivity during trauma exposure, which is positively associated with symptom severity. The mPFC is typically volumetrically smaller amongst patients with PTSD and is hypo-responsive during symptomatic states, with an inverse correlation to symptom severity (Shin et al., 2006). The insula may also have a critical role in PTSD. Resting state scans were used in one study to classify with 80% accuracy participants with PTSD versus non-clinical controls based on insula connectivity patterns (Harricharan et al., 2020). Structural studies also show that in patients with PTSD compared to controls, the premotor cortex (Rocha-Rego et al., 2012) insula (Kunimatsu et al., 2020), and hippocampus (Shin et al., 2006) have diminished volumes, with the hippocampus showing aberrant functional, and neuronal integrity (Shin et al., 2006). Newer findings also suggest diminished gray matter volume in the right dlPFC, OFC, and SMA in 30 females with PTSD based on childhood trauma versus controls (Thomaes et al., 2010).

When comparing the neural signature of misophonia and PTSD, similarities emerge in insula dysfunction. In resting state scans, participants with both misophonia and PTSD display PFC-insula hyperconnectivity. Furthermore, symptom provocation evidences hyperconnectivity between the insula and the amygdala as well as hyperactivity in the insula in both PTSD and misophonia. Nevertheless, PTSD seems to be characterized by reduced volumes in many of the structures of interest, a neural abnormality that has not been related to misophonia yet. Future studies should examine in more detail volumetric reductions in key brain regions in misophonia to further elucidate differences or similarities to PTSD. Furthermore, insula hyperactivity is not characteristic of PTSD dysfunction, but is characteristic of misophonia. Similarly, OFC dysfunction is characteristic of PTSD but not of misophonia. Thus, there appear to be several differences in PTSD and misophonia neural signatures to highlight the uniqueness of each disorder.

### Summary

Taken together (see Table 2), these findings highlight that, while there is overlap in neural dysfunction in misophonia and comorbid disorders, no other disorder can fully explain the alterations seen in misophonia. Of all comorbid disorders discussed here, PTSD and OCD neural dysfunction come closest to misophonia, although the hallmark of these disorders are the amygdala and the OFC, not the insula. When tasks relevant to the disorder examined are employed (e.g., symptom provocation, exposure to negative stimuli, emotion regulation in mood disorders, planning in OCD, working memory in ADHD), across comorbid disorders and misophonia, hyperactivity in the insula, amygdala, ACC, and hypoactivity in the dlPFC can be found. Therefore, the hyperactivity in subcortical regions seen in misophonia may be related to aberrant processing of, or hypersensitivity to, negative emotions. Furthermore, in misophonia and beyond, the dlPFC may be a general marker of problematic allocation of resources to respond to challenging contexts.

Findings related to the OFC and the motor cortex appear to be unique to misophonia. The dysfunction seen in the OFC appears to vary depending on the disorder under investigation with a unifying theme across comorbid disorders of functional hypoactivity and hypoconnectivity when disorder-relevant tasks are employed. The pattern of hyperconnectivity with other frontal and premotor regions appears to be uniquely related to misophonia. Across comorbid disorders, there was little examination of the role of the premotor cortex in psychiatric presentations. While hyperactivity in both SMA and PMv was found in misophonia during symptom provocation, other disorders were characterized by either unremarkable performance in these regions or by hypoactivity. Taken together, these findings strongly suggest a unique neural signature for misophonia, supporting an independent problem in need of novel personalized solutions.

## Neuroscience-informed interventions: Brain stimulation

In order to most rapidly identify an intervention for misophonia, a disorder for which there is currently no consensus for an optimal treatment [see Aazh et al. (2019) for a review of evidence for cognitive-behavioral interventions], neuroscientific dysfunctions that are unique to this problem should be directly targeted and altered. Thus, translating findings from basic neuroscience studies into innovative therapies can be the quickest way to finding a cure for misophonia. Non-invasive neurostimulation (i.e., the purposeful modulation of neural circuitry) is a powerful tool that resulted in novel interventions for several treatment resistant conditions, such as treatment refractory MDD (Neacsiu and Lisanby, 2015), OCD (Trevizol et al., 2016), smoking (Maiti et al., 2017), and PTSD (Kan et al., 2020).

Initially, neurostimulation targeted cortical regions at the surface of the brain which were reachable by the generated e-Field (which generally has a 2-cm depth of penetration below the scalp; Deng et al., 2013). Nevertheless, research findings suggest that the effect of neurostimulation can be seen throughout entire networks. A recent systematic review of over 33 rTMS studies found that active rTMS induces significant changes in resting state functional connectivity in a variety of targeted networks (Beynel et al., 2020). Furthermore, functional and structural networks can then be used to alter connectivity and activity in subcortical structures, such as the insula (Addicott et al., 2019) amygdala (Baeken et al., 2010), or ACC (Vink et al., 2018). In this way, neurostimulation can be used to remediate dysfunctional brain circuits regardless of their location.

### Overview of neurostimulation approaches

Because the brain is an electric organ, communications within neural networks may be altered with the use of magnetic and electric fields that induce brief activity in targeted brain cells. Magnetic stimulation relies on an electromagnetic coil, while electric stimulation passes direct current through the cortex in order to achieve neuromodulatory effects (Larrivee, 2020). In this section, we introduce several types of neurostimulation applications, focusing on those that have been successfully used in interventions for psychiatric disorders (see Table 3 for a summary).

**TABLE 3:** Overview, advantages, and risks of various neurostimulation techniques.

Technique	Overview	Advantages	Risks and disadvantages
Repetitive Transcranial Magnetic Stimulation (rTMS)	Uses a figure-8 coil to generate a magnetic field that induces electricity within brain region right underneath the center of the coil. RTMS uses trains of magnetic pulses at specific intervals called inter-train interval (ITI). Frequencies of stimulation lower than 5 Hz are considered inhibitory, while over 5 Hz are considered excitatory.	RTMS is the most traditional application of brain stimulation that has been FDA-approved for several interventions. There are several devices available that administer rTMS safely (Rossi et al., 2021) and a wide body of literature that characterizes parameter differences exists and can inform novel interventions.	RTMS can be painful or uncomfortable for up to 40% of those who undergo this treatment modality. There is a very low likelihood for seizures, especially with excitatory stimulation. Other risks are scalp, jaw, or face muscle contractions, mild headaches, and transient mood changes. Treatments that involve rTMS alone may require daily visits to a site where equipment to administer it exists.
Deep Transcranial Magnetic Stimulation (dTMS)	Uses an H-shaped coil, which is inserted in a spherical helmet placed on the head. The resulting magnetic field can induce electrical current in deeper brain regions than rTMS. The gain in depth comes with reduced stimulation precision.	Deeper structures, such as the medial prefrontal cortex or the anterior cingulate cortex, can be targeted using this technology. The use of a helmet to host the coil may make it easier to administer than rTMS.	Potential risk of dTMS are similar to rTMS with the addition of possible facial, tooth, or neck pain usually just during the stimulation.
Theta Burst Stimulation (TBS)	Uses a figure-8 coil, like rTMS but instead of trains of single pulses, delivers trains of triple pulses at a higher frequency.	The main advantage is that the same amount of stimulation achieved with a 35–40 min rTMS session can be achieved with only 3 min of iTBS. This allows for accelerated sessions (i.e., having multiple stimulation sessions in the same day)	The trade-off of increases efficiency of TBS comes with an increased risk for seizure. However, seizures are still considered a rare event.
Transcranial Direct Current Stimulation (tDCS)	Uses direct electrical currents to stimulate a brain network. Two electrodes placed over the head modulate neuronal activity *via* a steady current. Has two different types depending on need: anodal, which excites the network it targets, and cathodal, which reduces neuronal activity, thus allowing for greater control.	TDCS devices are much cheaper and easier to maintain than rTMS/dTMS/TBS devices. Naïve adults can be taught to use these devices in their own homes, increasing feasibility of dissemination. Furthermore, integration with MRI and EEG is easier to accomplish with tDCS than with other stimulation modalities.	The risks of tDCS are similar to those of rTMS. There is also a low probability for scalp burns. A disadvantage of this technology is that the results for its efficacy are mixed (e.g., Santos et al., 2018), and, therefore, it may be less effective than other types of neurostimulation. Currently, there is no FDA approved treatment that relies on tDCS, and experts highlight the need for more mechanistic understanding for this technology (Fregni et al., 2015).

Transcranial magnetic stimulation (TMS) employs rapidly alternating magnetic pulses that induce an electric current in the underlying cortex. It can be applied in single or repetitive pulses to either activate, enhance, or inhibit activation in a superficial neural target. The induced current depolarizes cortical neurons and alters the excitability of neural tissue (Neacsiu and Lisanby, 2015). High-frequency (HF; up to 20 Hz) rTMS has been associated with more excitability resulting in enhanced activity, and low frequency (≤1 Hz) with less excitability, and inhibited activity (Pascual-Leone et al., 1994; Chen et al., 1997; Neacsiu and Lisanby, 2015).

Repetitive TMS (rTMS) is the most traditional therapeutic application of brain stimulation (Neacsiu and Lisanby, 2015). It uses a figure-8 coil that is placed over a predetermined location of the head (called a target). The coil generates a magnetic field that induces electricity in the target by delivering magnetic pulses (or trains) on and off, at specific intervals inter-train intervals (ITI). An additional parameter important for rTMS is the motor threshold (MT). This parameter represents the lowest output of the rTMS machine needed to reliably elicit a motor movement when stimulating the motor area (i.e., the lowest output needed to reach the brain; Neacsiu and Lisanby, 2015). As an example, a typical rTMS treatment session for depression will contain 75 trains, each train being 4 s long, with an ITI of 26 s, delivered at 120% MT (Holtzheimer and McDonald, 2014). HF-rTMS has been FDA approved as a treatment for depression since 2008 (Holtzheimer and McDonald, 2014). Furthermore, HF-rTMS over the dlPFC and superior medial frontal cortex, has been found to significantly inhibit activity in the right insula (Li et al., 2017b; Chang D. et al., 2018). Therefore, rTMS is a successful approach to changing function in structures relevant to misophonia.

Deep transcranial magnetic stimulation (dTMS) aims to target “deeper” regions of the brain by using an H shaped coil pattern. This coil is inserted in a spherical helmet placed on the patient’s head. Generally, dTMS follows the same parameters as rTMS, with the difference being that dTMS is less precise in hitting its target. The FDA recently approved dTMS in conjunction with symptom provocation as a treatment for OCD in 2018 (Roth et al., 2021) and for smoking cessation in 2020 (Young et al., 2021). DTMS has also been employed in the treatment of PTSD (Isserles et al., 2021) and has evidence of successful use in interventions to target both cortical and subcortical structures (Beynel et al., 2021; Roth et al., 2021).

Intermittent theta burst stimulation (iTBS) is also delivered *via* a figure-8 coil, using triple pulses at a higher frequency to deliver unique, high-energy frequency of stimulation. The same effect that rTMS achieves with 75 trains, can be achieved with only 20 trains of iTBS, each train lasting 2 s, 8 s ITI. Therefore, iTBS session can be completed in about 3 min versus rTMS sessions which take 35–40 min (Pabst et al., 2022). Given that the majority of currently approved treatment protocols include 20–30 sessions [for depression for example (Sonmez et al., 2019)], iTBS can save significant time for patients. Recently, an iTBS protocol has obtained approval for treatment-resistant depression intervention (Blumberger et al., 2018). TBS has also been used successfully to alter amygdala activity *via* its connectivity with the STS (Pitcher et al., 2017), highlighting that this approach can successfully alter cortical and subcortical brain structures, including areas indicated in misophonia.

Transcranial direct current stimulation (tDCS) is an alternative type of brain stimulation that uses direct electrical currents to stimulate a specific brain network. A constant small current (1–2.5 mA) passes through two electrodes placed over the head in order to modulate neuronal activity. Anodal stimulation acts to excite the network it targets while cathodal stimulation reduces neuronal activity (Fregni et al., 2015). TDCS has not yet been cleared by the FDA in the treatment of psychiatric disorders because mechanisms through which tDCS operates need to be better understood (Fregni et al., 2015). Nevertheless, it is widely researched for its therapeutic applications, because of the low cost and ease to administer. TDCS interventions are thought to be probably efficacious for non-drug resistant depression (Lefaucheur et al., 2017), currently unsuccessful with tinnitus (positive response for 15% of 602 participants across six studies; Santos et al., 2018), and potentially relevant for remediating aberrant amygdala hyperactivity (Ironside et al., 2019).

Neurostimulation interventions have primarily been developed and successful for adults with psychiatric disorders who did not respond to other treatments. Meta-analyses of therapeutic applications within these samples find effect sizes that are small-to-moderate when compared to placebo (Neacsiu and Lisanby, 2015). Findings suggest that these effect sizes could be improved by using connectivity-driven targeting (Fox et al., 2012; Opitz et al., 2016; Fox, 2018), neuroimaging and neuro-navigation (Neacsiu and Lisanby, 2015; Beynel et al., 2019) and employing electric field (E-field) modeling (Bungert et al., 2017; Opitz et al., 2016). In addition, fusing neuromodulation with behavioral practice (Tsagaris et al., 2016) can enhance efficacy. For example, combining HF-rTMS with active emotion regulation practice yields behavioral improvements in emotion regulation up to a week after a single session when compared to emotion regulation practice alone in transdiagnostic clinical adults (Neacsiu et al., 2022b,a). In addition, combinations of rTMS with 16–20 sessions of psychotherapy demonstrate feasibility (Neacsiu et al., 2018), with enhanced effects over psychotherapy alone (Kozel et al., 2018), or over cognitive training alone (Cunningham et al., 2015).

Both researchers and consumers should be aware of the potential risks involved with neurostimulation. The most severe, but rare, risk is for seizure, which in prior studies occurred in about 0.2% of research subjects (Rossi et al., 2021). The most common side effects are headaches or muscle tension which can occur in up to 30% of those receiving neurostimulation, depending on the protocol and target (Rossi et al., 2009). Discomfort at the site of neurostimulation during the procedure can happen in up to 40% of patients (Rossi et al., 2009). Nevertheless, less than 2% of research participants quit because of pain and discomfort, and the majority of participants experienced habituation to this discomfort over time (Rossi et al., 2009). Other, less common, side effects are temporary changes in mood, dizziness, and hearing impairment. ’Another potential risk is that compensatory rather than dysfunctional networks may be impacted by this particular treatment. Several guidelines have been established to guide development and application of neurostimulation interventions in order to maximize safety (Rossi et al., 2009, Rossi et al., 2021).

In addition to risks, it is important to highlight that there continue to be many unknowns with regards to this technology. There is limited data on the long-term effects of neurostimulation interventions (Marangell et al., 2007) and the parameter space (frequency, intensity, coil positioning, and orientation; Beynel et al., 2019). Furthermore, the exact mechanism through which neurostimulation changes psychopathology in the brain is still unknown (Bestmann and Feredoes, 2013). Other types of neurostimulation exist, such as transcranial photobiomodulation, alternating current stimulation, or focused ultrasound. While these technologies can also offer promise to treatments in general, they will not be discussed in this review because of their limited existing evidence for broad therapeutic effects in psychiatric disorders. Therefore, researchers and clinicians interested in neurostimulation approaches should continue to follow the literature for best practices and new approaches to increase safety and efficacy.

### Neurostimulation as a treatment for misophonia: design considerations

A neurostimulation treatment for misophonia could be constructed through several avenues. On the one hand, based on resting state misophonia findings, one could develop a neurostimulation intervention that attempts to reduce hyperconnectivity between IFC-TPJ (Eijsker et al., 2021b), PMv-insula, or other such hyperactive circuits (Kumar et al., 2021). In order to reduce hyperconnectivity, inhibitory neurostimulation may be attempted as a first choice of intervention. Existing studies highlight that inhibitory neurostimulation can be successfully applied over the TPJ (Powers et al., 2020a) and the PMv (Tremblay et al., 2012). To enhance the efficacy of neurostimulation, resting state functional imaging data should be collected prior to treatment administration in order to identify the regions within the TPJ and the PMv that are connected to IFC and the insula, respectively. These regions should then be exposed to repetitive inhibitory neurostimulation using either rTMS or continuous TBS (Huang et al., 2005). Accelerated neurostimulation (Baeken, 2019), or several consecutive sessions should be examined to determine the optimal amount of neurostimulation to elicit changes in misophonia.

On the other hand, given the therapeutic synergy between behavioral and neuromodulatory interventions, one could alter context either before or during neuromodulation. One approach would be to expose participants to misophonic triggers before administering neurostimulation, similar to the FDA approved paradigm for OCD using dTMS (Roth et al., 2021). The rationale behind the “symptom provocation” is that it activates the circuitry involved in misophonia leaving these networks more amenable to change. This provocation could be followed with excitatory neurostimulation targeted toward regions that downregulate the amygdala, the insula, or the ACC (such as the dlPFC or MPFC), as well as inhibitory neurostimulation over regions like the SMA, PMv, STG, or vmPFC. An alternative approach would be to expose participants to trigger sounds during neurostimulation. Options for brain stimulation while sounds are being played would be similar as previously described (e.g., excitatory over dlPFC/mPFC, or inhibitory over SMA/PMv/vmPFC/STG).

An additional option, following research in emotion regulation (Neacsiu et al., 2022a), would be to coach the use of an emotion regulation skill [e.g., distancing (Powers and LaBar, 2019)] while misophonic triggers are presented. Excitatory neurostimulation could then be concurrently administered over a node of the emotion regulation network (e.g., the dlPFC). Alternatively, inhibitory neurostimulation could be administered concurrently over a hyperactive area in misophonia (e.g., SMA). Yet another option could be to enhance dlPFC or SMFG activation using HF-rTMS during a stop signal task, to correct differences from healthy subjects found in misophonia (Eijsker et al., 2019).

While regions like the dlPFC or mPFC are mentioned several times as options for stimulation, the specific target within the dlPFC/mPFC might vary depending on the rationale for its selection. For low resource approaches, the structural dlPFC could be identified using the beam method (Beam et al., 2009), a targeting approach that uses scalp measurements to identify the optimal stimulation spot. A more precise approach would involve neuroimaging, either structural, to identify regions with more anatomic specificity, or functional, to identify specific dysfunctional networks that may be candidates for neurostimulation. For example, one might expose participants to trigger versus neutral sounds in the scanner. Using neuroimaging analysis software, the next step would be to compute a contrast in activation between these two different auditory experiences. For surface targets, the region with the highest activation within the vmPFC or within the PMv within this contrast could be extracted. For connectivity targets, the highest activation within the amygdala, insula, or ACC could be extracted, followed by an analyses to help identify a surface region with functional connectivity to one of these subcortical regions of specific activity.

Similar approaches have been successfully used in other disorders. For example, in adolescents with MDD, decreased amygdala volumes were normalized using HF-rTMS to the left dlPFC (Seewoo et al., 2022). Furthermore, HF rTMS to the dlPFC was found to significantly increase ACC activity (Tremblay et al., 2012), and resting state left dlPFC – ACC connectivity (Huang et al., 2005), and decrease right insula activity (Li et al., 2017a). In MDD and PTSD patients, 5 Hz TMS to the dlPFC was also found to reduce the problematic hyperconnectivity between the sgACC, the insula and the DMN (Philip et al., 2018). In borderline personality disorder, HF-rTMS over the right dlPFC yielded a decrease in connections between the amygdala/insula and precuneus, PCC, and parietal lobules (Sverak et al., 2021). Similarly, tDCS over the left dlPFC led to increased long-term cerebral blood flow to the ACC (Jog et al., 2021), and reduced activation of the amygdala (Ironside et al., 2019). Therefore, the dlPFC is a successful target for changing activity and connectivity of subcortical structures such as the insula and the ACC. These changes occur independent of the type of targeting employed, although more precise, connectivity-driven targeting is likely to have a more powerful effect than anatomically driven targeting (Neacsiu et al., 2018).

Different regions of the mPFC and TPJ have also been targeted successfully with documented downstream effects. For the treatment of OCD, after rTMS to the right OFC, PET scans revealed decreased metabolism in the ACC (Nauczyciel et al., 2014), implying deactivation in this region. Furthermore, 5 Hz rTMS over the mPFC led to an increase in functional connectivity between the mPFC and amygdala (Beynel et al., 2021). In MDD adults, HF rTMS over the dmPFC led to reduced dmPFC – insula and sgACC-caudate connectivity (Salomons et al., 2014). TDCS between the left TPJ and left PFC reduced TPJ-insula and TPJ-SMA functional connectivity in schizophrenia patients (Mondino et al., 2016) and in non-clinical adults (Dalong et al., 2021). Taken together, these studies offer insight into the feasibility and initial parameter set up necessary to engage other cortical targets in neurostimulation intervention.

Researchers are encouraged to examine the efficacy and optimal parameters necessary for misophonia interventions that utilize neurostimulation alone, or in conjunction with a behavioral intervention (see Table 4 for examples of the parameters to use.). It is important to highlight that the possible avenues for intervention presented in this paper are by no means exhaustive. Researchers should continue to examine the therapeutic potential of altering other circuits and brain regions as new theoretical findings and refined hypotheses emerge.

**TABLE 4:** Examples of specific parameters that are based on research findings or other protocols where similar goals (e.g., reducing hyperconnectivity) were accomplished for different types of neurostimulation discussed in this review.

Protocol type	Examples of parameters recommended by other experimental studies	Citation
Inhibitory rTMS	1 Hz, continuous 110% rMT	Tremblay et al., 2012 Turi et al., 2021
cTBS	Continuous train of 600–1,200 pulses applied in the theta burst pattern (bursts of three stimuli at 50 HZ repeated at 5 Hz frequency) 80% rMT, 600 total pulses	Huang et al., 2005 Valchev et al., 2015 Dutta et al., 2021
Inhibitory tDCS	Constant current of 1.5 mA	Antonenko et al., 2017
Excitatory rTMS	10 Hz, 4–5 s trains, 15 s inter-train intervals at 120% rMT, over 1,600 pulses	Horvath et al., 2010 Cash et al., 2017 Turi et al., 2021 Maeda et al., 2000
iTBS	Triplet 50 Hz bursts, repeated at 5 Hz; 200 ms on and 8 s off	Pitcher et al., 2017 Blumberger et al., 2018
Excitatory tDCS	Constant current of 1.5 mA	Baeken et al., 2010 Feeser et al., 2014

In conclusion, a novel neurostimulation intervention could be an effective way to help sufferers. For example, rTMS clinics are available in all 50 states, and over 250 million Americans have insurance plans that cover this approach to treatment (Neurostar, 2021). TDCS equipment is affordable and accessible, which has led to exciting innovations in administering tDCS interventions remotely, by sending devices at home and teaching patients how to independently use them (Charvet et al., 2015; Hordacre, 2018). In addition, TBS is emerging as the most efficient way to administer neurostimulation in a very short amount of time, allowing for massed sessions (Chung et al., 2016; Pabst et al., 2022). Furthermore, the funding for neurostimulation is skyrocketing, with hundreds of millions of dollars being raised worldwide to fund technology advancements (Albert, 2020). In short, neurostimulation is becoming the frontier for developing novel treatments worldwide and misophonia treatment research should harness this enthusiasm.

## Author contributions

AN conceptualized the review, summarized the research on misophonia, and wrote the section on neurostimulation treatment considerations. The co-authors each contributed several paragraphs to the other sections of the review. All authors contributed to the article and approved the submitted version.

## Conflict of interest

The authors declare that the research was conducted in the absence of any commercial or financial relationships that could be construed as a potential conflict of interest.

## Publisher’s note

All claims expressed in this article are solely those of the authors and do not necessarily represent those of their affiliated organizations, or those of the publisher, the editors and the reviewers. Any product that may be evaluated in this article, or claim that may be made by its manufacturer, is not guaranteed or endorsed by the publisher.

## Footnotes

1 It is important to highlight that misophonia *per se* was not measured in this study.
