# Supplementary material for: The neurobiology of misophonia and implications for novel, neuroscience-driven interventions
Source: Front Neurosci. 2022 Jul 25;16:893903. doi: 10.3389/fnins.2022.893903 (PMC9359080; doi:10.3389/fnins.2022.893903)
Supplement: Supplementary file 1 [file Table_1.docx]

Supplementary Table 1. Differences between misophonic adults and controls in neural activation across studies.

| **Study** | **Participants** | **Stimuli** | **Result** | **Brain Region** | **MNI Coordinates** |
| --- | --- | --- | --- | --- | --- |
| Ventral pre-motor cortex | | | | | |
| Kumar et al., 2021 | 20 misophonic adults and 22 healthy controls | 15 s aversive, neutral, and misophonic sounds | Misophonia group> control for aversive sounds only | Right ventral premotor cortex | 60, 12, 24 |
| Cerliani and Rouw, 2020 | 19 misophonic adults and 20 controls | 12 s trigger, aversive, and neutral | Misophonia group; trigger stimuli > aversive | Right ventrolateral premotor cortex | 49, 10, 2 |
| Insula | | | | | |
| Kumar et al., 2017 | 20 misophonic adults and 22 healthy controls | 15 s trigger, unpleasant, and neutral sounds | Misophonia group> control for trigger sounds only | Left anterior insula | -41, 6, 0 |
| Kumar et al., 2017 | 20 misophonic adults and 22 healthy controls | 15 s trigger, unpleasant, and neutral sounds | Misophonia group> control for trigger sounds only | Left dorsal anterior insula | -33, 23, 3 |
| Kumar et al., 2017 | 20 misophonic adults and 22 healthy controls | 15 s trigger, unpleasant, and neutral sounds | Misophonia group> control for trigger sounds only | Right anterior insula | 39, 23, -3  35, 30,0 |
| Schroder et al., 2019 | 21 misophonic adults and 23 controls | 25 s neutral, misophonic, and neutral sounds | Misophonia group > controls misophonic condition > neutral condition | Right insula | 32, 12, -14 |
| Cerliani & Rouw, 2020 | 19 misophonic adults and 20 controls | 12 s trigger, aversive, and neutral | Misophonia group; trigger stimuli > aversive | Dorsal anterior insula | 41, 10, 2 |
| Inferior frontal gyrus | | | | | |
| Kumar et al., 2017 | 20 misophonic adults and 22 healthy controls | 15 s trigger, unpleasant, and neutral sounds | Misophonia group> control for trigger sounds only | Left inferior frontal gyrus | -51, 2, 11 |
| Kumar et al., 2017 | 20 misophonic adults and 22 healthy controls | 15 s trigger, unpleasant, and neutral sounds | Misophonia group> control for trigger sounds only | Right inferior frontal gyrus | 48, 11, 2 |
| Supplementary motor area | | | | | |
| Kumar et al., 2017 | 20 misophonic adults and 22 healthy controls | 15 s trigger, unpleasant, and neutral sounds | Misophonia group> control for trigger sounds only | Right supplementary motor area | 5, 8, 66  5, -3, 68 |
| Cerliani and Rouw, 2020 | 19 misophonic adults and 20 controls | 12 s trigger, aversive, and neutral | Misophonia group; trigger stimuli > aversive | Bilateral supplementary motor area | -12, -2, 67  9, -1, 65 |
| Cerebellum | | | | | |
| Kumar et al., 2017 | 20 misophonic adults and 22 healthy controls | 15 s trigger, unpleasant, and neutral sounds | Misophonia group> control for trigger sounds only | Right cerebellum | 32, -63, -23  35, -56, -27  21, -68, -47  32, -56, -50  27, -65, -54 |
| Kumar et al., 2017 | 20 misophonic adults and 22 healthy controls | 15 s trigger, unpleasant, and neutral sounds | Misophonia group> control for trigger sounds only | Left cerebellum | -30, -65, -53  -27, -65, -24  -36, -57, -26  -45, -63, -29 |
| Cingulate cortex | | | | | |
| Kumar et al., 2017 | 20 misophonic adults and 22 healthy controls | 15 s trigger, unpleasant, and neutral sounds | Misophonia group> control for trigger sounds only | Left cingulate sulcus | -3, 27, 33 |
| Schroder et al., 2019 | 21 misophonic adults and 23 controls | 25 s neutral, misophonic, and neutral sounds | Misophonia group > controls; misophonic > neutral condition | Right anterior cingulate cortex | 4, 44, 16 |
| Cerliani and Rouw, 2020 | 19 misophonic adults and 20 controls | 12 s trigger, aversive, and neutral | Misophonia group; trigger stimuli > aversive | Mid-cingulate cortex | 11, 14, 38 |
| Auditory cortex | | | | | |
| San Giorgi, 2015 | 10 misophonic adults and 7 health controls | 25 s misophonic, aversive, and neutral sounds | Misophonia group>controls; misophonic >  Neutral sounds | Right auditory cortex | 54, -26, 2  66, -12, 0 |
| San Giorgi, 2015 | 10 misophonic adults and 7 health controls | 25 s misophonic, aversive, and neutral sounds | Misophonia group; misophonic > neutral sounds | Left auditory cortex | -54, -34, 10 |
| Amygdala | | | | | |
| San Giorgi, 2015 | 10 misophonic adults & 7 healthy controls | 25 s misophonic, aversive, and neutral sounds | Misophonia group > controls; misophonic > aversive sounds | Left amygdala | -16, -2, -12 |
| San Giorgi, 2015 | 10 misophonic adults & 7 healthy controls | 25 s misophonic, aversive, & neutral sounds | Controls > misophonia group; aversive sounds | Left amygdala | -16, -2, -14 |
| Frontal Gyrus | | | | | |
| Kumar et al., 2017 | 20 misophonic adults and 22 healthy controls | 15 s trigger, unpleasant, and neutral sounds | Misophonia group > control; trigger sounds only | Left middle frontal gyrus | -38, 39, 21 |
| Kumar et al., 2017 | 20 misophonic adults and 22 healthy controls | 15 s trigger, unpleasant, and neutral sounds | Misophonia group > control; trigger sounds only | Left superior frontal gyrus | -50, 12, 29 |
| Temporal cortex | | | | | |
| Schroder et al., 2019 | 21 misophonic adults and 23 controls | 25 s neutral, misophonic, and neutral sounds | Misophonia group > controls; misophonic > neutral sounds | Right superior temporal cortex | 60, -26, 6 |
| Schroder et al., 2019 | 21 misophonic adults and 23 controls | 25 s neutral, misophonic, and neutral sounds | Controls > misophonia group | Right inferior temporal gyrus | 44, -62, -12 |
| Kumar et al., 2017 | 20 misophonic adults and 22 healthy controls | 15 s trigger, unpleasant, and neutral sounds | Misophonia group> control; trigger sounds only | Left supramarginal gyrus | -59, -41, 29 |
| Other regions | | | | | |
| Kumar et al., 2017 | 20 misophonic adults and 22 healthy controls | 15 s trigger, unpleasant, and neutral sounds | Misophonia group > control; trigger sounds only | Left thalamus | -11, -23, -11 |
| Kumar et al., 2017 | 20 misophonic adults and 22 healthy controls | 15 s trigger, unpleasant, and neutral sounds | Misophonia group> control; trigger sounds only | Right precentral gyrus | 48, 5, 39  47, 2, 53 |
| Schroder et al., 2019 | 21 misophonic adults and 23 controls | 25 s neutral, misophonic, and neutral sounds | Controls > misophonia group | Right fusiform gyrus | 34, -78, -14 |
| Cerliani and Rouw, 2020 | 19 misophonic adults and 20 controls | 12 s trigger, aversive, and neutral | Misophonia group; trigger stimuli > aversive | Visual area V1/V2 | 20, -70, 21 |
| Cerliani and Rouw, 2020 | 19 misophonic adults and 20 controls | 12 s trigger, aversive, and neutral | Misophonia group; trigger stimuli > aversive | Frontal right operculum | 48, 9, 1 |
| Kumar et al., 2017 | 20 misophonic adults and 22 healthy controls | 15 s trigger, unpleasant, and neutral sounds | Misophonia> control for trigger sounds only | Right brain stem | 9, -30, -11  9, -41, -44  5, -45, -47 |
